# Cannabis Consumption Among Musicians: About A Series Of 37 Cases

**DOI:** 10.1192/j.eurpsy.2023.1414

**Published:** 2023-07-19

**Authors:** S. Brahim, W. Bouali, I. Ghachem, M. Kacem, S. Khouadja, R. Ben Soussia, S. Younes, L. Zarrouk

**Affiliations:** 1 psychiatry; 2 Neurology, Taher Sfar, Mahdia, Tunisia

## Abstract

**Introduction:**

Many musicians have suffered the consequences of drug addiction. What about cannabis use?

**Objectives:**

To describe the epidemiological characteristics of cannabis users among musiciens

To study the prevalence of anxiety and depression disorders among these consumers

**Methods:**

A descriptive and retrospective study of the epidemiological characteristics and prevalence of depression and anxiety in a population of 37 musicians who consume cannabis. This sample was selected among 202 musicians having participated in an anonymous questionnaire

**Results:**

The prevalence of cannabis use among musicians in our study is about 18.31%. 76% of them are professionnals with sex ratio of 6.25. The mean age of these musicians is 27 years old. They started using cannabis at a mean age of 21 years old. The history of school failure was found in 1/3 of all cases, with a younger age at the onset of cannabis use (18 years old vs 22 years old in absence of school failure). 72.4% of cannabis users are single, 27.5% are in a relationship, 66.7% of the 37 musicians are Tabaco smokers, 55.6% are alchoolics, and 19,4% are using other drugs. 16.7% of these musicians are followed for depressive disorder, anxiety or bipolar disorder. The mean duration of cannabis use is 7 years, often in group of people. The first contact with cannabis occurs after the start of learning music in 44.4% of cases (a mean of 12 years after).The average consumption is about 4 times per week, mostly outside the musical activity in 3/4 of the cases. 53.6% believe that cannabis can cause a decline in their health. 10 musicians increased cannabis use and 8 of them believe that it can improve their performance and creativity. On the other hand, only 9 musicians wish to wean the use of cannabis. 19/29 musicians (65.5%) have an anxiety (A) and/or depression (D), that is proven to be moderate to severe respectivelly in 2/3 and half of cases, The mean of the A score and D score of the HAD scale is 10 and 9, respectivelly.

**Conclusions:**

The reasons of cannabis addiction are various: fleeing reality, seeking the anxiolytic or sedatif effects and improving performance.

**Disclosure of Interest:**

None Declared

